# Peer Review of “Willingness to Pay for the COVID-19 Vaccine and Its Correlates in Bangladesh: Cross-Sectional Study”

**DOI:** 10.2196/79355

**Published:** 2025-08-15

**Authors:** Enamul Kabir

**Affiliations:** 1 University of Southern Queensland, UniSQ Toowoomba, 487-535 West St, Queensland, Australia, 61 1800 269 500

**Keywords:** Bangladesh, willingness to pay, vaccines, COVID-19, infectious diseases, infection control, public health, public safety, cross-sectional study, financial

*This is a peer-review report for “Willingness to Pay for the COVID-19 Vaccine and Its Correlates in Bangladesh: Cross-Sectional Study.”*

## Round 1 Review

This paper [1] addresses an important and timely topic—willingness to pay for COVID-19 vaccines in a developing country context. Understanding willingness to pay is essential not only for informing current vaccine financing strategies but also for shaping policies related to equitable vaccine access in response to future public health challenges. The study is well-conceived and provides valuable insights into vaccine affordability and public perception in Bangladesh. With some refinements in presentation, statistical interpretation, and policy framing, the paper will be well-positioned for publication.

The abstract would benefit from being more concise and should more clearly highlight the key policy implications of the findings. Additionally, the statistical interpretation of the adjusted odds ratios (aORs) requires careful attention. Several aORs are reported with values close to 1 (eg, family income aOR 1.0, *P*=.039; vaccine knowledge aOR 1.1, *P*=.003; behavioral practices aOR 1.1, *P*<.001), suggesting minimal effect sizes, yet they are statistically significant. While such significance may be driven by the large sample size, reporting CIs would allow for a more meaningful interpretation of the strength and direction of these associations.

The paper would also benefit from greater clarity around the construction of variables and the underlying measurement models. It is unclear how multiple survey items were combined to form factors such as knowledge, attitudes, and behavioral constructs. Using exploratory factor analysis could be beneficial to validate the grouping of items into coherent factors and strengthen construct validity. Providing factor loadings or at least a brief description of the item-grouping process would enhance the methodological transparency of the study.

Another area for improvement involves the reporting of the income variable. In both Table 1 and Table 2, income appears to be modeled as a continuous variable, but the unit of measurement is not specified. Without this information, it is difficult to interpret an odds ratio of 1.0 meaningfully. If income is measured in small units (eg, Bangladeshi taka), the impact of each unit increase would be negligible. Categorizing income into meaningful brackets (eg, low, middle, high) and using those categories in logistic regression would make the results more interpretable and policy relevant. Additionally, the CIs for some variables in Table 2—such as income and COVID-19 vaccine conspiracy beliefs—appear to suggest nonsignificance, yet they are reported as significant. This inconsistency should be carefully reviewed and clarified.

Some of the measured constructs, such as knowledge and perceived susceptibility, show relatively low internal consistency (eg, Cronbach α of 0.612 and 0.657, respectively). It would be helpful for the authors to explain why these values are considered acceptable in this context or to discuss efforts made to improve reliability through item refinement or scale revision. Furthermore, the combination of nonprobability online sampling and quota sampling should be more clearly justified. While practical during a pandemic, it raises concerns about representativeness and potential sampling bias, which should be acknowledged more explicitly in the Discussion.

The manuscript would also benefit from a thorough review for minor language and formatting issues. For instance, the phrase “explains explains” on page 13 should be corrected. Variable labels and descriptions in tables should be presented clearly and consistently.

Overall, this is a valuable study that contributes to the growing body of literature on COVID-19 vaccine access and health economics. With revisions to enhance clarity, statistical reporting, and methodological transparency, the paper has strong potential for publication and meaningful policy impact.

## Abbreviations

aOR: adjusted odds ratio
